# NPs-TiO_2_ and Lincomycin Coexposure Induces DNA Damage in Cultured Human Amniotic Cells

**DOI:** 10.3390/nano9111511

**Published:** 2019-10-23

**Authors:** Filomena Mottola, Concetta Iovine, Marianna Santonastaso, Maria Luisa Romeo, Severina Pacifico, Luigi Cobellis, Lucia Rocco

**Affiliations:** 1 Department of Environmental, Biological and Pharmaceutical Sciences and Technologies, University of Campania “Luigi Vanvitelli”, 81100 Caserta, Italy; filomena.mottola@unicampania.it (F.M.); concetta.iovine@unicampania.it (C.I.); maui35@hotmail.it (M.L.R.); severina.pacifico@unicampania.it (S.P.); 2 Department of Woman, Child and General and Special Surgery, University of Campania “Luigi Vanvitelli”, 80138 Napoli, Italy; marianna.santonastaso@unicampania.it (M.S.); luigi.cobellis@unicampania.it (L.C.); 3 Sant’ Anna e San Sebastiano Hospital, 81100 Caserta, Italy

**Keywords:** titanium dioxide nanoparticles, lincomycin, human amniotic cells, in vitro genotoxicity, apoptosis, DNA damage

## Abstract

Titanium dioxide nanoparticles (NPs-TiO_2_ or TiO_2_-NPs) have been employed in many commercial products such as medicines, foods and cosmetics. TiO_2_-NPs are able to carry antibiotics to target cells enhancing the antimicrobial efficiency; so that these nanoparticles are generally used in antibiotic capsules, like lincomycin, added as a dye. Lincomycin is usually used to treat pregnancy bacterial vaginosis and its combination with TiO_2_-NPs arises questions on the potential effects on fetus health. This study investigated the potential impact of TiO_2_-NPs and lincomycin co-exposure on human amniocytes *in vitro*. Cytotoxicity was evaluated with trypan blue vitality test, while genotoxic damage was performed by Comet Test, Diffusion Assay and RAPD-PCR for 48 and 72 exposure hours. Lincomycin exposure produced no genotoxic effects on amniotic cells, instead, the TiO_2_-NPs exposure induced genotoxicity. TiO_2_-NPs and lincomycin co-exposure caused significant increase of DNA fragmentation, apoptosis and DNA damage in amniocytes starting from 48 exposure hours. These results contribute to monitor the use of TiO_2_-NPs combined with drugs in medical application. The potential impact of antibiotics with TiO_2_-NPs during pregnancy could be associated with adverse effects on embryo DNA. The use of nanomaterials in drugs formulation should be strictly controlled in order to minimize risks.

## 1. Introduction

The toxicology and safe application of the recently developed nanoparticles (NPs) have raised great interest in the last years. There is a growing demand on the use of these NPs in different industries due to some physicochemical properties: small size, large surface area, redox potential, photocatalytic and quantum properties [1,2,3,4]. NPs can easily be released and enter human body during the use of commercial products. It has emerged that all types of NPs tested are able to cross the placental barrier [5,6,7]. Notably, the pregnant women cannot avoid exposing to them. Several studies have shown toxicity and genotoxicity of a wide range of engineered nanoparticles [8,9,10,11,12,13], consequently this aspect has raised concerns regarding the health of exposed organisms. Among nanomaterials, titanium dioxide nanoparticles (TiO_2_-NPs) are the most commonly used. TiO_2_-NPs are applied in medicine as photosensitizer for photodynamic therapy [2,14], drug delivery [15,16] biomedical ceramics [17] and implant biomaterials [18], in foods [19], in cosmetics as sunscreen, toothpaste and personal care products [20,21,22], in sterilization and in paint industry [23,24]. NPs-TiO_2_ are inert and poorly soluble nanoparticles that can be absorbed by living organisms generally by oral ingestion, this is the main way of absorption as they are used a food additive, in toothpaste, capsules, and various foods [25]. Regarding the extensive application of TiO_2_-NPs in everyday life, the question arises as to whether this nanoparticle has detrimental effects on human health. Human exposure to TiO_2_-NPs may also occur through inhalation and ingestion, and then penetrate into the circulatory system and reach other organs (liver, spleen, lungs, brain and testis) [26,27,28]. TiO_2_-NPs exposure causes ovarian and female reproductive system dysfunction in mice [29]. These nanoparticles, like all types of tested NPs, are able to cross the placental barrier and induce damage [6]. TiO_2_-NPs prenatal exposure could increase the risk of gestational diabetes, in fact nanoparticles increase maternal fasting blood glucose levels due to gut microbiota alterations [30]. Some studies have also shown that TiO_2_-NPs can be transferred from pregnant mice to their offspring and affect mice hippocampus by degeneration, necrosis, and the absence of axonal outgrowth of offspring neurons. This suggested that maternal exposure to TiO_2_-NPs caused learning and memory decline in offspring by decreasing the number of neurons and inhibiting axonal and dendritic outgrowth of hippocampal neurons [31]. Furthermore, the TiO_2_-NPs induce behavioral deficits related to autism spectrum disorder and neurodevelopmental disorders [32]. The gestational exposure to TiO_2_-NPs impairs the growth and development of placenta in mice with a mechanism that seems to be involved in vascularization, proliferation and apoptosis pathways [33]. These alterations cause pregnancy complications, fetal growth retardation and adverse birth outcomes [34], associated with embryonic and bone toxicity due to TiO_2_-NPs accumulation in fetal mice. These effects may be due to the direct or indirect role of TiO_2_-NPs interfering with Ca, Zn, and other metabolic processes [35]. NPs-TiO_2_ induce cytotoxicity and reduce mitotic index in human amniotic fluid cells perturbing cells adhesion ability [36]. One of the most worrying characteristics of NPs-TiO_2_, as for all NPs, is their ability to carry any type of substances to the target cells, thus determining a greater effectiveness of the molecule carried, which does not spread throughout the body, but acts directly on the intended target. At the same time, the transport of a toxic substance could improve its toxicity and/or genotoxicity (“Trojan horse” effect) [13,37,38,39,40]. The substances, potentially carried by NPs, can be environmental pollutants such as heavy metals or even drugs. This effect is also used in medicine to deliver drugs to target organs. In fact, one approach for increasing the antimicrobial efficacy of antibiotics, without raising the overall dose, is to increase the local targeting concentration by conjugating antibiotics with nanoparticles [41,42]. So, the application of nanoparticles has emerged as an option in the control of bacterial infections in many drugs, that present titanium dioxide as a coloring additive of the capsule, such as lincomycin.

Lincomycin and its derivatives (clindamycin) are antibiotics widely used in clinical practice for the treatment of bacterial infections, in particular those caused by anaerobic species. Lincomycin is bacteriostatic, inhibiting protein synthesis in sensitive bacteria (especially Gram-positive and also protozoans), and bactericidal if used at higher concentrations [43]. Lincomycin and clindamycin are usually used in treatment for bacterial vaginosis in pregnancy, where the normal vaginal microbiota is replaced by a mixed anaerobic microbiota. Bacterial vaginosis may be associated with an increased risk of spontaneous preterm delivery and other complications during pregnancy [44,45], and then the treatment is necessary.

Given the importance of maternal and fetus health during pregnancy, investigation on possible effect of TiO_2_-NPs on amniotic fluid cells will give important information about TiO_2_-NPs biohazards [36,46]. Therefore, the amniotic cells have been chosen as a model in this study with the aim to assess the effects of titanium dioxide nanoparticles and lincomycin. Lincomycin has been used alone and in combination with TiO_2_-NPs to demonstrate the ability of nanoparticles to influence the antibiotic action and also to detect the possible “Trojan horse” effect. Cytological effects and genotoxic damage have been evaluated through the trypan blue vitality test, Comet and Diffusion Assay and the RAPD-PCR technique for two exposure times (48 and 72 h).

## 2. Materials and Methods

### 2.1. Chemicals

Titanium dioxide nanoparticles (Aeroxide) were supplied by Evonik Degussa (Essen, Germany; Lot. 614061098). Aeroxide has been 99.9% pure certified and is a blend of 75% rutile and 25% anatase forms with a dimensional average of 21 nm. The preparation of the TiO_2_-NPs stock solution (10.0 mg/L) was performed according to literature data [13,38]. Briefly, TiO_2_-NPs solution underwent, ultrasonication to disperse nanoparticles and to eliminate agglomeration. Sonication was carried out in medium (Millipore) for 3 h (40 kHz frequency, Dr. Hielscher UP 200S, Germany). UV–Vis spectra were acquired in the range of 200–600 nm by a Shimadzu UV-1700 double beam spectrophotometer. No absorption was detected in the range 300–400 nm, where characteristic peaks for TiO_2_-NPs nanoparticles aggregates are known to appear [11,13]. Lincomycin (CAS 7179-49-9, 99% purity) was provided from Sigma-Aldrich. This product was provided as delivered and specified by the issuing Pharmacopoeia. All the substances tested were dissolved in DMSO (dimethylsulfoxide, CAS. 67-68-5).

### 2.2. Chromatographic Analysis

Chromatographic analysis was carried out using an Agilent 1260 Infinity II HPLC system equipped with 1260 Infinity II VL quaternary pump, and 1260 Infinity II DAD WR diode array detector. CDS LC ChemStation OpenLAB Software was used for data acquisition and analysis.

Separation was achieved using Phenomenex Luna Phenyl-Hexyl, 150 × 2 mm i.d. column (3.0 μm particle size) using a gradient of water (A) and acetonitrile (B), both with 0.1% formic acid. Starting with 10% B, a linear gradient was followed to 25% B in 6.0 min, and held at 25% B for other 1.0 min. Finally, starting conditions were restored and the system re-equilibrated for other 1 min. The total analysis time was 8.0 min, the flow rate was 0.3 mL min^−1^. Injection volume was 5.0 μL.

### 2.3. Cell Culture and Exposure Procedure

The human amniotic cells were collected from amniotic fluid of pregnant women undergoing prenatal diagnosis for possible chromosomal abnormalities as a routine procedure during the mid-trimester (15–18 weeks of gestation) at Sant’ Anna e San Sebastiano Hospital (Caserta, Italy). Amniotic fluid was collected from pregnant women after written informed consent which was obtained from all participants or their legal guardians, in compliance with the Declaration of Helsinski. Amniotic fluid samples were centrifugate at 1500 rpm for 10 min and suspended into a medium specific for amniocytes growth (Amniomed ® Plus, EuroClone). This medium is a complete medium which contains L-glutamine, FBS, phenol red, sodium bicarbonate, antibiotics and all the necessary growth factors for optimal and selective amniocytes growth. The conditions for the proliferation of amniocyte clones (temperature: 37 °C; pH: between 7.2–7.4; CO_2_: 5%) onto a plastic culture flask (surface of 25 cm^2^) are selected according to Ascar and collaborator, 2015 [36]. The obtained primary culture amniotic cell was tripsinized, harvested for metaphase spreads and analysed for prenatal diagnosis. After performing prenatal diagnosis, 32 secondary culture amniotic cell (sub-cultures) were then allowed to expand until clones >50 cells formed reaching the cell confluence. Clones were pooled, centrifuged at 1500 rpm for 10 min and suspended into medium and replated on plastic culture flask. When the sub-cultures reached the confluence, the cells were trypsinized with 1 mL of 1 X trypsin-EDTA (Microgem Cat. L0930-100) and divided in four experimental groups: one flask treated with 10 μg/L of TiO_2_-NPs, one with 100 mg/L of lincomycin, one with 10 μg/L of TiO_2_-NPs plus 100 mg/L of lincomycin, and the last one with 20 µl of DMSO as negative control. As a positive control, 10 mM of H_2_O_2_ was used. We used a single concentration of TiO_2_-NPs antibiotic according to our previous genotoxic studies [11,47]. Time exposure were 48 and 72 h. The same cells pool has been used across all assays and experiments. Incubation was performed as described above. All experiments were performed in triplicate.

### 2.4. Viability Assay

Amniotic cell viability was assessed by blue trypan assay according to literature data [48]. A cell suspension was mixed with 0.4% dye and examined on a slide with optical microscope to discriminate cells that incorporate the dye from cells that exclude it. A viable cell will have a clear cytoplasm whereas a nonviable cell will have a blue cytoplasm.

### 2.5. Comet Assay

DNA strand breaks in human amniotic cells have been evaluated by Comet assay [49]. The Comet assay provided the trypsinization of cell samples and then centrifugation at 2000 rpm for 5 min, the pellet was re-suspended in 200 μL of physiological solution. So, the amniotic cells have been mix with the Low Melting Point Agarose (0.5%) and were included into Normal Melting Agarose (1%) layers on slides. After overnight incubation in the cold lysis solution (NaCl 2.5 M, Na_2_EDTA 0.1 M, Tris-Base 0.4 M, TRITON-X100 1%, DMSO 10%, pH 10), the slides were site for 10 min in alkaline buffer (NaOH 10N, EDTA 200 mM, pH 12.1), then were exposed to electrophoresis (25V,300 mA) for 15 min. Finally, the slides were fixed in cold methanol, stained with 30% ethidium bromide and observed by the fluorescence microscope with 60X magnification (Nikon Eclipse E-600). Comet assay was performed in triplicate. The images were acquired by means of the “OpenComet” software [50]. The parameter considered was the percentage of damaged DNA present in the comet tail (% Tail DNA). Highly damaged cells, also known as ghost cells or clouds, have been excluded from analysis because they artifactually increase the apparent DNA fragmentation due to actual genotoxicity. In fact, the ghost cells detected after the treatments were due both to the genotoxic damage and to the apoptosis induced by these substances, so they do not allow to discriminate the two processes [51].

### 2.6. Diffusion Assay

Diffusion assay protocol is the same as the Comet Assay, but the cell slides do not undergo electrophoresis. This assay showed the apoptotic cells that are characterized by irregular contours with nuclei with highly dispersed DNA. The nuclei of the necrotic cells, on the other hand, are larger and not well defined [52]. Diffusion assay was performed in triplicate. The Diffusion assay slides were scored by subdividing the degree of DNA diffusion pattern in five classes of damage as reported by Cantafora and collaborator in 2014 [53] and we considered only class 5 (apoptotic cell).

### 2.7. RAPD-PCR Technique

Amniotic cell DNA was isolated from 200 µl of all treated cell suspension using the High pure PCR template preparation Kit (ROCHE Diagnostics) according to the manufacturer’s instructions to guarantee a sufficiently pure extraction to produce a good quality RAPD-PCR profile. The amplification DNA protocol was conducted through primer 6 (5′-d[CCCGTCAGCA]-3′) [54]. The amplification program provides one first step at 94 °C for 2 min, then 1 min at 95 °C, 1 min at 36 °C and 2 min at 72 °C, for 45 cycles. The reaction products were analysed by means of electrophoresis on 1,5% agarose gel and examined after gel staining with 1% ethidium bromide. The RAPD-PCR profiles have polymorphic patterns that allow the calculation of the template genomic stability (GTS%) as follows:

GTS = (1 − a/n) × 100

where *a* is the average number of polymorphic bands found in each treated sample and *n* is the total number of bands in the negative control. Appearance of new bands and disappearance of bands are polymorphism detected in RAPD-PCR profiles, which are analysed by comparison to the control sample. The average is calculated for each sample exposed to different molecules. The variations of these values are estimated as a percentage respect to the control set to 100% [13].

### 2.8. Statistical Analysis

The data were expressed as mean and standard deviation (SD). Differences in the percentage of cell viability, DNA damage and apoptosis genomic stability among the experimental groups were analyzed with the unpaired Student’s t-test using GraphPad Prism 6. Only results with p-value ≤ 0.05 were considered statistically significant.

## 3. Results

### 3.1. Characterization and Analytical Determinations

**Figure 1:**
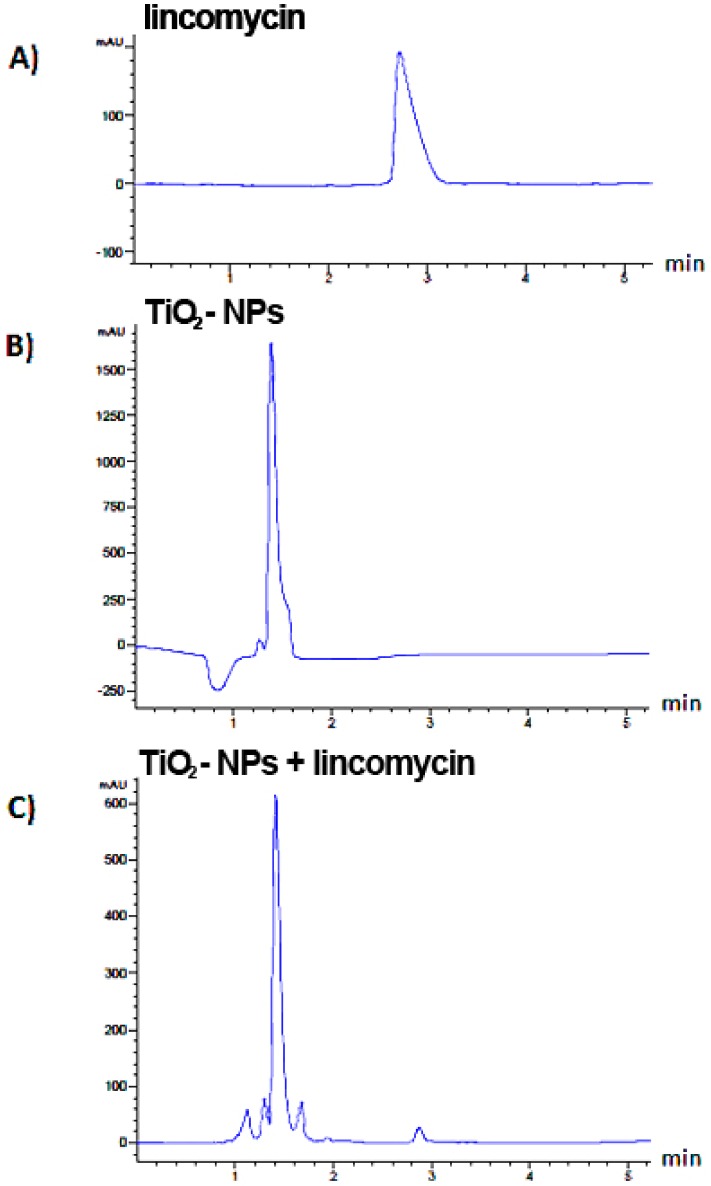
Representative chromatograms of lincomycin, TiO_2_-NPs and TiO_2_-NPs + −72h.

Culture media respectively enriched in lincomycin, TiO_2_-NPs and TiO_2_-NPs combined with lincomycin underwent chromatographic separation (Figure 1) and UV spectra for chromatographic peaks were extracted. The spectrum of pure lincomycin showed a weak absorbance, with a maximum at 195 nm.

**Figure 2:**
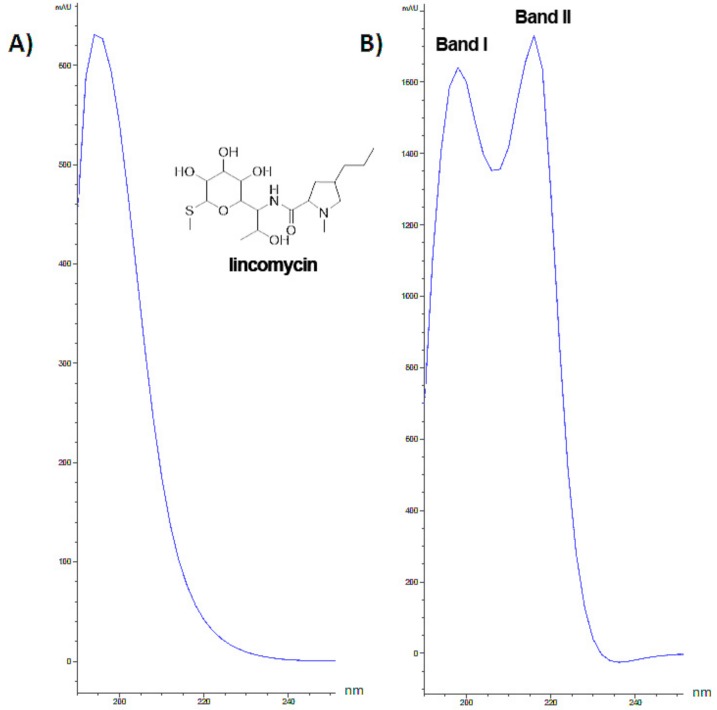
Extracted UV spectra of lincomycin (**A**), and TiO_2_-NPs (**B**) in the range 190–_2_50 nm.

In order to verify TiO_2_-NPs and lincomycin interactions, TiO_2_-NPs absorbance was also acquired in the range 190–250 nm. Two bands were detected, the first one (herein described as Band I) was at 196 nm, whereas the second (Band II) at 240 nm (Figure 2).

**Figure 3:**
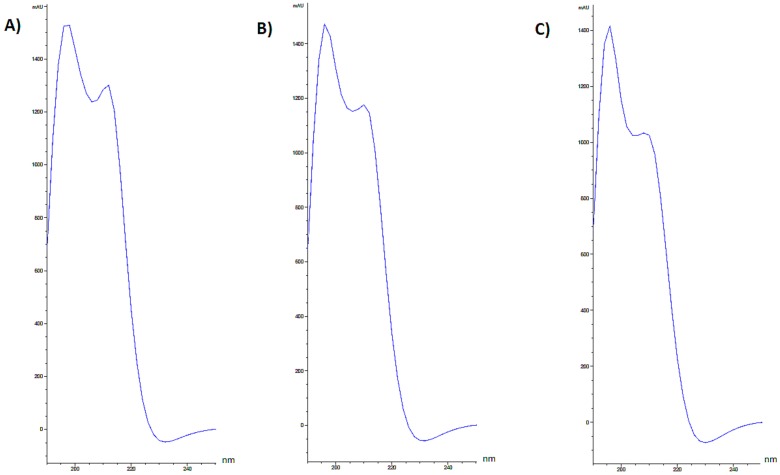
Extracted UV spectra of TiO_2_-NPs + lincomycin in the range 190–250 nm at (A) t = 0h; (B) t = 48h; (C) t = 72h.

Extracted UV spectra of TiO_2_-NPs and lincomycin combination showed a hyperchromic effect. In fact, a single peak was detect, with a retention time almost superimposable to that of TiO_2_-NPs. Indeed, it was observed that a time-dependent increase of TiO_2_-NPs Band I absorbance (Figure 3).

### 3.2. TiO_2_-NPs Reduces Amniotic Cells Viability

**Figure 4:**
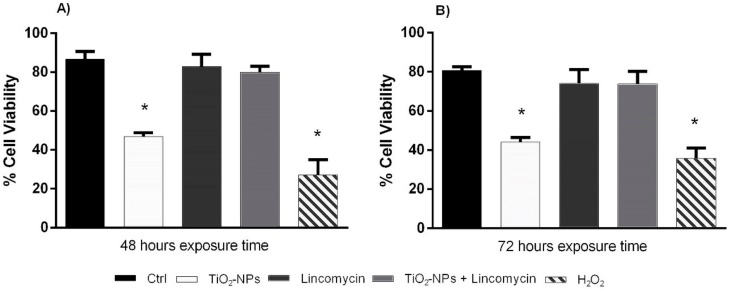
Percentage of alive amniotic cells (ordinate) after 48 h exposure time (**A**) and after 72 h exposure time (**B**) to TiO_2_-NPs, lincomycin and their combination (abscissa). The dark bar is negative control; the white bar is 10 μg/L TiO_2_-NPs treated cells; the dark grey bar is 100 mg/L lincomycin treated cells; the light grey bar is 10 μg/L TiO_2_-NPs + 100 mg/L lincomycin treated cells; the striped bar is 10 mM H_2_O_2_ treated cells. * *p* ≤ 0.05.

48 and 72 exposure hours to TiO_2_-NPs statistically significant reduced amniotic cell viability. The exposure to TiO_2_-NPs plus lincomycin did not induce a statistically significant changes in viability (Figure 4).

### 3.3. TiO_2_-NPs and Lincomycin Co-Exposure Induces an Increase of Amniocyte DNA Fragmentation

**Figure 5:**
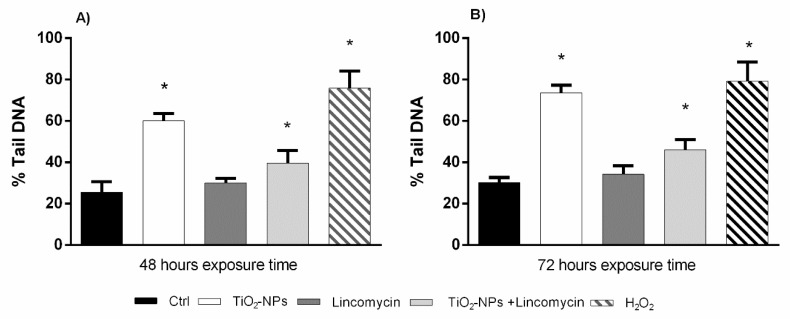
Percentage of DNA in the tail of the comet in amniotic cells (ordinate) after 48 h exposure time (**A**) and after 72 h exposure time (**B**) to TiO_2_-NPs, lincomycin and their combination (abscissa). The dark bar is negative control; the white bar is 10 μg/L TiO_2_-NPs treated cells; the dark grey bar is 100 mg/L lincomycin treated cells; the light grey bar is 10 μg/L TiO_2_-NPs + 100 mg/L lincomycin treated cells; the striped bar is 10 mM H_2_O_2_ treated cells. * *p* ≤ 0.05.

The results from Comet Assay showed a statistically significant increase of cell DNA fragmentation after 48 and 72 h exposure to TiO_2_ nanoparticles alone and in combination with lincomycin. The exposure to lincomycin did not show statistically significant DNA damage (Figure 5).

### 3.4. TiO_2_-NPs in Combination with Lincomycin Cause Amniotic Cells Apoptosis

**Figure 6:**
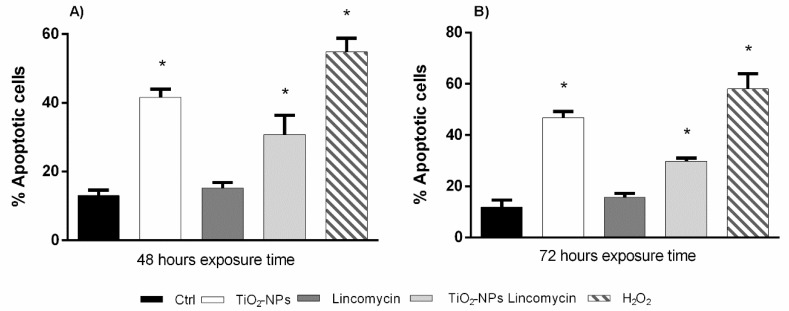
Percentage of apoptotic cells after (ordinate) after 48 h exposure time (**A**) and after 72 h exposure time (**B**) to TiO_2_-NPs, lincomycin and their combination (abscissa). The dark bar is negative control; the white bar is 10 μg/L TiO_2_-NPs treated cells; the dark grey bar is 100 mg/L lincomycin treated cells; the light grey bar is 10 μg/L TiO_2_-NPs + 100 mg/L lincomycin treated cells; the striped bar is 10 mM H_2_O_2_ treated cells. * *p* ≤ 0.05.

The data from Diffusion assay did not show statistically significant apoptotic damage induced by lincomycin. The exposure to TiO_2_-NPs and co-exposure to TiO_2_-NPs and lincomycin after 48 and 72 h induced a statistically significant increase of apoptotic amniotic cells (class 5) with respect to the negative control (Figure 6).

### 3.5. TiO_2_-NPs and Lincomycin Determines a Change of DNA Polymorphic Profiles

The RAPD-PCR analysis showed a variation of the polymorphic profiles of the amniotic cell DNA exposed to TiO_2_-NPs and lincomycin after 48 and 72 exposure hours respect to the DNA of the not-treated amniotic cells. 48 h exposure to lincomycin and TiO_2_-NPs induced the variation of one and two bands respect to control polymorphic profiles respectively. Co-exposure to lincomycin and TiO_2_-NPs induced the loss of two bands respect to the control. 72 h exposure to lincomycin determined the gain of a band, instead TiO_2_-NPs treatment caused three bands variation; lincomycin and TiO_2_-NPs combination showed the variation of two bands (Table 1).

### 3.6. TiO_2_-NPs and Lincomycin Co-Exposure Decreases Amniotic Cells Genomic Stability

**Figure 7:**
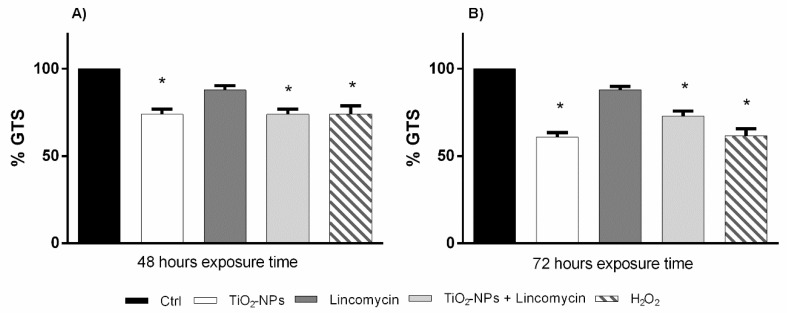
Changes in percentage of Genome Template Stability (ordinate) in amniotic cell DNA after 48 h exposure times (**A**) and after 72 h exposure time (**B**) to TiO_2_-NPs, lincomycin and their combination (abscissa). The dark bar is negative control; the white bar is 10 μg/L TiO_2_-NPs treated cells; the dark grey bar is 100 mg/L lincomycin treated cells; the light grey bar is 10 μg/L TiO_2_-NPs + 100 mg/L lincomycin treated cells; the striped bar is 10 mM H_2_O_2_ treated cells. * *p* < 0.05.

The polymorphic profiles obtained by RAPD-PCR were used to evaluate the percentage of genome stability in human amniotic cells exposed to TiO_2_-NPs alone and in combination with lincomycin *in vitro*. 48- and 72 h exposure to TiO_2_ nanoparticles and co-exposure with lincomycin induced a statistically significant reduction in genomic stability (Figure 7).

## 4. Discussion

The nanoparticles large-scale application has raised the attention on the possible adverse effects on the exposed organism’s health. Titanium dioxide is among the most used nanoparticles in different industrial sectors. NPs-TiO_2_ can penetrate in different internal organs through inhalation and ingestion and consequently accumulate inducing DNA damage, ROS production, apoptosis and change in cell cycle and nuclear membranes [55,56,57,58]. TiO_2_-NPs are able to diffuse through the protective cellular barriers, such as a placental barrier, and may also involve risks to human health. On that regard it is needed an in-depth investigation of their possible toxicological effects. In an *ex-vivo* human placental perfusion model, Wick and collaborators [59] demonstrated the uptake of nanosized fluorescently labeled polystyrene beads of 50, 80, 240, and 500 nm across the placental barrier. Also, in animal models, the translocation of TiO_2_-NPs has been reported in brain of prenatally exposed mice. Since the blood barriers are underdeveloped in the fetus, the nanoparticles could easily pass into brain during the early stages of fetal development [60]. In the study conducted by Saquib and collaborators [46], the DLS data revealed the formation of TiO_2_-NPs aggregates in the RPMI cell culture medium, which were also found to be internalized in the TEM images of the treated human amniotic cell lines. These aggregated NPs, in culture medium, have been reported to enter into cells, such as human amniotic cells, mainly through endocytosis. Their localization in the vacuoles and cell cytoplasm of the exposed cells corresponds well with the observations of Hussain and collaborators [61,62]. In order to safeguard and protect pregnant women and fetus health, the knowledge of the toxic/genotoxic mechanisms of NPs-TiO_2_ and their association with drugs is very important. The unnecessary supplementation of drugs and commercial products with TiO_2_-NPs should be restrained during pregnancy until its detrimental effects on embryo development have been clarified. The present work investigated the genotoxic effect of titanium dioxide-NPs and lincomycin on human amniotic fluid cells *in vitro*. The results show that lincomycin exposure has no toxic/genotoxic effects on amniotic cells; differently, TiO_2_-NPs exposure induced an increase of DNA strand breaks, a reduction of cells viability, a loss of DNA stability and apoptosis for each time tested (48–72 h). Each type of cell has its own vulnerability to NPs; in fact, the metabolic rate, the antioxidant enzyme machinery and DNA repair capabilities of each cell type responded differently based on the concentration of TiO_2_-NPs, which induced different toxicity levels. Moreover, several crystalline forms of NP-TiO_2_ and the aggregation of NPs determined a change in their effects due to the increase in their size. Sadhukha and collaborators [63] showed how particle size is a determining factor in biological functions, demonstrating how well-dispersed nanoparticles induce apoptosis while aggregates show different effects.

In order to protect the cell, we suggest that DNA repair activity would be activated as a consequence of DNA damage in cells exposed to TiO_2_ nanoparticles. This response could be different related to the exposure time and dosage. The results of this study are in accordance with Boland and collaborators [64] who only investigated the effect of short-term exposure to TiO_2_-NPs and demonstrated how titanium dioxide nanoparticles are harmful to cell survival by activating an exuberant apoptotic response. The latter, to be activated, it requires the internalization of the TiO_2_ nanoparticles since the accumulation in lysosomes leads to their rupture and to the release of hydrolases such as cathepsins and intracellular ROS production. The primary mechanism of NPs induced toxicity is due to oxidative stress, resulting in damage to cellular membranes and biological macromolecules [65,66,67,68]. Our results have demonstrated that the TiO_2_-NPs induced DNA damage in amniocytes at a concentration of 10 μg/L. Thus, the dose comparison of our study with earlier reports [69,70] suggests the induction of oxidative stress in human amniotic derived fluid cells at relatively lesser concentrations of TiO_2_-NPs. Furthermore, the nanomaterials released in the cytoplasm allow their access to essential biomolecules causing damage. In addition, the nanoparticles can reach the nucleus, in fact, a high fragmentation was also found by Patel and collaborators in 2017 [71]. The authors showed that exposure to ·OH significantly increases damage to the DNA, which was quantified as a moment and as a percentage of DNA in the tail, in peripheral blood lymphocytes. NPs-TiO_2_ could induce ·OH radicals which are probably responsible for the DNA damage in the exposed cells [72]. There are contradicting results in literature on exposure with different NPs at different stages of embryo development and also with the differences in experimental models [73,74,75]. This raises a concern for pregnant women who could be exposed unconsciously due to the environmental spread of these nanoparticles, in fact, one of the main exposures for the general population is through food consumption products, even from food packaging [76]. This study investigation shows that the co-exposure TiO_2_-NPs and lincomycin induced genotoxic effects without any cytotoxic one; in fact the co-exposure times affected the cell survival and induced changes in genetic material which do not cause cytotoxic effects. However, this mechanism has not been clarified and needs further investigations. Human amniocyte co-treated with TiO_2_-NPs and lincomycin showed the increase of DNA fragmentation, induction of apoptotic process and DNA damage. The results of Comet Assay demonstrate that the co-exposure induced a statistically significant increase of DNA strand breaks starting from 48 exposure hour. The data obtained by Diffusion assay highlighted the increase of amniotic cell apoptosis from 48 to 72 exposure hours after TiO_2_-NPs and lincomycin co-exposure. Similarly, the DNA genomic stability was statistically reduced after the co-exposure.

However, we stress that although co-exposure induces genotoxic effects, the latter are still lower than the exposure to TiO_2_-NPs alone. The results of this work suggested that the combination of TiO_2_-NPs and lincomycin cause an intermediate level of genotoxicity to amniocytes compared to the exposure of each single compound which by themselves are quite toxic to these cells. Nevertheless, the combination tested produce less toxicity that the same quantity of NPs probably because the co-exposure could affect the bioavailability of the NPs or their biophysical characteristics (e.g., aggregability). In fact, extracted UV spectra of lincomycin and TiO_2_-NPs showed that when media with TiO_2_-NPs combined with lincomycin was injected, in each chromatogram, a single peak was detected and its retention time seemed almost superimposable to that of TiO_2_-NPs. The observation of this hyperchromic effect, together with the disappearance in the HPLC chromatogram of the peak related to pure lincomycin, allowes us to hypothesize the interactions have been established. Therefore, the results demonstrate that the combination of TiO_2_-NPs and lincomycin leads to the aggregate formation reducing the genotoxcity compared with the dispersed nanoparticles.

The loss or gain of genotoxicity drugs and/or pollutants carried by titanium dioxide nanoparticles could depend on the molecule carried and media, determining or not the “Trojan horse effect”. However, the experimental evidence on “Trojan horse effect” is conflicting, in fact, it has been demonstrated no interaction of the TiO_2_-NPs with organic pollutants (CdCl_2_ and dioxin) in *Dicentrarchus labrax* in sea water [38,39]. Instead, TiO_2_-NPs reduced CdCl_2_-induced effects and DNA damage in *Mytilus galloprovincialis*, whereas, additive effects were no observed [31]. According to these reports, our results suggest the absence of synergistic effect after co-exposure to TiO_2_-NPs and lincomycin on human amniotic cells, rather co-exposure reduces the TiO_2_-NPs genotoxicity. Considering that Trojan horse effect is controversial, it could be hypothesized that these nanoparticles could influence the activity of the lincomycin in such a way that could improve its efficacy and thus lead to dosage reduction.

The exposure to TiO_2_-NPs during pregnancy should be thoroughly investigated and exposure to NPs must be prevented or minimized. Nevertheless, it must be considered that the behavior of the substances tested is relative to an in vitro system that use a single concentration substances, so, it is necessary to complement the in vitro testing by performing a complete genotoxicity assay on a large range of concentrations and by accurately assessing the level of cytotoxicity; then, by performing methodologies able to display relevant genetic events (mutagenesis, chromosomal aberrations). In this way, a complete in vitro genotoxicity profile will be available. As a result, further studies on other in vivo models could be considered to clarify this aspect, so as to carry out prevention interventions on maternal exposure. The data assume that the genotoxicity studies of NPs must be recommended during a comprehensive assessment of the safety of TiO_2_-NPs and novel types of NPs and nanomaterials.

## Figures and Tables

**Table 1:** Molecular sizes (bp) of appeared and disappeared bands after amplification with primer P6 in amniotic cell DNA exposed to the TiO_2_-NPs and lincomycin. *Control bands are at: 200, 300, 320, 400, 500, 520, 700, 950 bp.

Substances Concentration	Hours of Exposure	Gained Bands *	Lost Bands *
TiO2-NPs 10 µg/L	48 72	850, 530 650	- 320, 400
Lincomycin 100 mg/L	48 72	- 650	400 -
TiO2-NPs 10 µg/L + Lincomycin 100 mg/L	48 72	- 650	400, 520 300
